# Rise and fall of outbreak-specific clone inside endemic pulsotype of *Salmonella* 4,[5],12:i:-; insights from high-resolution molecular surveillance in Emilia-Romagna, Italy, 2012 to 2015

**DOI:** 10.2807/1560-7917.ES.2018.23.13.17-00375

**Published:** 2018-03-29

**Authors:** Marina Morganti, Luca Bolzoni, Erika Scaltriti, Gabriele Casadei, Elena Carra, Laura Rossi, Paola Gherardi, Fabio Faccini, Norma Arrigoni, Anna Rita Sacchi, Marco Delledonne, Stefano Pongolini

**Affiliations:** 1 Istituto Zooprofilattico Sperimentale della Lombardia e dell'Emilia-Romagna, Sezione di Parma, Parma, Italy; 2 Istituto Zooprofilattico Sperimentale della Lombardia e dell'Emilia-Romagna, Risk Analysis Unit, Parma, Italy; 3 Istituto Zooprofilattico Sperimentale della Lombardia e dell'Emilia-Romagna, Sezione di Modena, Modena, Italy; 4 Local Health Unit of Piacenza, Department of Public Health, Piacenza, Italy; 5 Istituto Zooprofilattico Sperimentale della Lombardia e dell'Emilia-Romagna, Sezione di Piacenza, Gariga-Podenzano, Italy

**Keywords:** *Salmonella enterica* 4,[5],12:i:-, clone, pulsed-field gel electrophoresis, Multiple- Locus VNTR Assay, Whole genome sequencing, salami, outbreak

## Abstract

Epidemiology of human non-typhoid salmonellosis is characterised by recurrent emergence of new clones of the pathogen over time. Some clonal lines of *Salmonella* have shaped epidemiology of the disease at global level, as happened for serotype Enteritidis or, more recently, for *Salmonella* 4,[5],12:i:-, a monophasic variant of serotype Typhimurium. The same clonal behaviour is recognisable at sub-serotype level where single outbreaks or more generalised epidemics are attributable to defined clones. The aim of this study was to understand the dynamics of a clone of *Salmonella* 4,[5],12:i:- over a 3-year period (2012–15) in a province of Northern Italy where the clone caused a large outbreak in 2013. Furthermore, the role of candidate outbreak sources was investigated and the accuracy of multilocus variable-number tandem repeat analysis (MLVA) was evaluated. **Methods:** we retrospectively investigated the outbreak through whole genome sequencing (WGS) and further monitored the outbreak clone for 2 years after its conclusion. **Results:** The study showed the transient nature of the clone in the population, possibly as a consequence of its occasional expansion in a food-processing facility. We demonstrated that important weaknesses characterise conventional typing methods applied to clonal pathogens such as *Salmonella* 4,[5],12:i:-, namely lack of accuracy for MLVA and inadequate resolution power for PFGE to be reliably used for clone tracking. **Conclusions**: The study provided evidence for the remarkable prevention potential of whole genome sequencing used as a routine tool in systems that integrate human, food and animal surveillance.

## Introduction

Salmonellosis is among the most frequently reported food-borne diseases worldwide [1] and the second most-reported in the European Union (EU) [2]. In the past few decades, the epidemiology of salmonellosis has seen the emergence and/or expansion of specific types of *Salmonella* both at serotype and sub-serotype level [3]. In some instances, these newly-emerged types have gained wide and long-lasting geographic diffusion, as in the case of serotype Enteritidis, which gained worldwide epidemic diffusion in the 1980s and 1990s [4]. More recently, *Salmonella* 4,[5],12:i:- has been among the most frequently isolated serotypes from human cases in the EU [5-8]. The clonality of these emerging *Salmonella* has challenged the routinely-used typing methods and the effectiveness of laboratory-based surveillance [9,10].

In this study, we retrospectively investigated a large outbreak by *Salmonella* 4,[5],12:i:- through whole genome sequencing (WGS) after it had become evident that conventional epidemiology and routine molecular methods, namely PFGE and multilocus variable-number tandem repeat analysis (MLVA), could not elucidate some critical aspects of the infectious episode. Furthermore, to accurately understand the temporal dynamics of the outbreak clone we extended WGS monitoring of the clone from 1 year before the outbreak onset to 2 years after its conclusion, i.e. from June 2012 to December 2015. The outbreak, which occurred in the Emilia-Romagna region of Italy in 2013, was detected by the routine regional surveillance system, based on PFGE. The epidemiological investigation rapidly identified the food involved (fermented dry-cured salami made from pork) and the facility implicated, but was not able to attribute several individuals infected with the implicated strain to the outbreak and could not confirm or exclude the role of suspect sources at abattoir and farm level. This was mainly the consequence of the outbreak pulsotype (STYMXB.0131) being the most common in the surveillance database and endemic in the area since at least 2008 [8,11]. More specifically, in the monitored pre-outbreak period (June 2012 to June 2013) the pulsotype represented 8.9% of all *Salmonella* isolates from humans and 19.2% of *Salmonella* 4,[5],12:i:-, in line with previous reports of this pulsotype in several European countries since the mid-2000s [12,13]. In particular, an extended Swiss study reported 37% prevalence of the pulsotype among human isolates of *Samonella* 4,[5],12:i:- over the period 2007–11 [14]. Additional uncertainty during the outbreak investigation originated from the finding of variant types, although similar, among outbreak-related isolates, by PFGE (one variant) and, even more, by MLVA (five variants). The study was designed to address the unresolved issues and to answer specifically the following questions: (i) was there an outbreak-specific clone inside the endemic pulsotype STYMXB.0131 of *Salmonella* 4,[5],12:i:- and was it limited to the outbreak or circulating before and after it?; (ii) what was the exact responsibility of different candidate sources of the outbreak?; (iii) to what extent did the many observed MLVA variants belong to the outbreak clone? In other words, how accurate is MLVA in tracking *Salmonella* Typhimurium and variants in field conditions?; and (iv) what lessons can be learnt about the benefit of WGS as a surveillance tool in field conditions?

## Methods

### Laboratory surveillance system for salmonellosis

The laboratory surveillance system for human salmonellosis for Emilia-Romagna, an administrative region in Northern Italy with a population of ca 4.5 million residents [15], is a routine system based on serotyping and PFGE typing of all isolates recovered weekly from the regional network of medical microbiology laboratories. MLVA is performed on all isolates of *S.* Typhimurium and *Salmonella* 4,[5],12:i:- at a later stage (1–3 weeks later), as supplementary information to PFGE. To promptly identify potential outbreaks, a weekly analysis of the surveillance database is performed to assess whether any of the pulsotypes shows clustering in time beyond the expected threshold as previously described [16,17]. The outbreak alarm statistic is calculated with the OutbreakP algorithm from the ‘surveillance’ package in the R statistical environment [18]. The algorithm uses a weekly rolling window to evaluate whether the number of cases belonging to a given pulsotype is significantly greater than the expected number of cases during that period, based on the database of isolates starting from June 2012.

The system is integrated with the regional animal and foodstuff surveillance, which includes testing of official samples, private diagnostic activity on animals and some self-control testing by the food industry.

### Epidemiological investigation

Case definition: following the alert for a possible outbreak of salmonellosis, a case was initially defined as any culture-confirmed human infection by *Salmonella* 4,[5],12:i:- with pulsotype STYMXB.0131, detected in the province of Piacenza after 22 July 2013. For the purpose of this study, the case definition included cases up to the epidemiologically defined end of the outbreak set at 29 October 2013. No cases other than laboratory-confirmed ones were considered within the scope of the study.

Case interviews: the cases were interviewed by staff from the Local Health Unit of Piacenza using a standard format from the food-borne outbreaks reporting manual in accordance with directive 2003/99/EC from 2011 [19].

### Microbiological investigation

Various types of samples were collected from the suspected sources during the outbreak investigation and tested for *Salmonella*. Sampling included salami, pork, the surface of pig carcasses, swine faeces, environmental swabs and faeces from the workers at the implicated salami-production facility. Foodstuffs were tested according to Regulation 2073/2005 [20]; for carcasses, four different sites of 100 cm^2^ each (hind limb medial, back, belly and cheek) were sampled using the abrasive sponge method. Swine faeces and boot swabs were tested with ISO 6579:2002/Amd.1: 2007 [21]. After the end of the outbreak, intensified monitoring of the salami-production facility was implemented within its hazard analysis and critical control points (HACCP) plan. The post-outbreak monitoring, from November 2013 to December 2015, included 123 batches of salami, 87 batches of raw meat, and 83 environmental swabs.

### Typing of isolates and phylogenetic analysis

After serotyping, the monophasic character of the isolates was confirmed by PCR as previously described [22]. PFGE was performed according to the PulseNet protocol with *XbaI* digestion of DNA [23] and restriction profiles were analysed by BioNumerics 7.5 (Applied Maths, Saint-Martens – Latem, Belgium). MLVA was performed as previously described [24] with a CEQ 8000 Genetic Analysis System (Beckman Coulter, US) and profiles were assigned according to the MLVA nomenclature suggested by Larsson et al. (2009) [25].

Ninety-eight isolates of the outbreak-related pulsotypes underwent WGS (Table 1), comprising a set of 19 diverse STYMXB.0131 isolates presumed to be unrelated to the outbreak and included in the analysis as outgroup isolates. To maximise the probability of these isolates not being outbreak-related, and therefore constituting adequate outgroup isolates, they were selected from human, animal and food isolates recovered from other parts of the region excluding Piacenza. They included a cluster of six isolates representing a small known outbreak that occurred in a nursery school 200 km from Piacenza in November 2012.

**Table 1:** Isolates of *Salmonella* 4,[5],12:i:-, study in Emilia-Romagna, Italy, 2012–2015 (n = 198)

Source	Recovered isolates	WGS-analysed isolates	MLVA-variant isolates^a^		PFGE-variant isolates^b^	
			Number of isolates	Number of types	Number of isolates	Number of types
Human (during putative outbreak period)	137	35^c^	5	4	0	0
Human (outside putative outbreak period)	21	21	13	7	0	0
Salami production facility and salami	14	14	6	2	3	1
Suppliers of salami production facility	26	9^d^	5	2	7	1
Outgroup isolates	NA	19	15	7	0	0
**Total**	**198**	**98**	**44**	**NA**	**10**	**NA**

MLVA: multilocus variable-number tandem repeat analysis; NA: not applicable; WGS: whole genome sequencing.

^a^ With reference to outbreak type 3–13–9-NA-211; all variant isolates were WGS analysed.

^b^ With reference to outbreak pulsotype STYMXB.0131; all variant isolates were WGS analysed.

^c^ Isolates selected for WGS analysis were evenly distributed throughout the putative outbreak period.

^d^ Including seven randomly selected STYMXB.0083 isolates of the 24 recovered from pig farm and the two STYMXB.0131 isolates recovered from pig farm and external slaughterhouse.

For WGS, sequencing libraries were prepared from genomic bacterial DNA with the Nextera XT sample preparation kit and run on an Illumina MiSeq (Illumina, San Diego, US) with a 2x250 paired-end run. The reads of the 98 genomes of *Salmonella* 4,[5],12:i:- were deposited at the European Bioinformatics Institute under Project Number PRJEB7560. The average sequencing coverage was 194X with > 75% of bases having Q value equal to or greater than 30. The genome of outbreak isolate STM45, used as reference for phylogenetic analysis, was assembled with MIRA 4.0 [26] using ‘accurate settings for de novo assembly mode’ and discarding small contigs (size < 1,000 bp). The average assembled-genome characteristics were: 5,010,485 nt length, 158X coverage, 144 contigs > 5,000 nt and N50 of 77,805.

For phylogenetic analysis, the single nucleotide polymorphisms (SNPs) of each genome were extracted using a reference-based United States Food and Drug Administration SNP-pipeline (version 0.6.1), based on Bowtie2 and VarScan [27]. The default settings of the pipeline were used for SNP calling, i.e. a minimum coverage of 8x and a minimum variant allele frequency of 0.90. A Bayesian tree was generated with MRBAYES [28] from the SNP matrix of the analysed genomes. The Bayesian analysis was run using the GTR substitution model for 2,000,000 generations with chain sampled every 1,000th generation. The final parameter values and trees were summarised after discarding 25% of the posterior sample.

We assessed whether the occurrence of outbreak isolates with variant MLVA increased as a function of the isolation date by fitting a generalised linear model (GLM) with binomial error distribution. Similarly, we assessed whether the number of SNPs to the consensus, in the polymorphic positions shared by all outbreak-related human isolates, increased as a function of the isolation date by fitting a GLM with Poisson error distribution.

### Antimicrobial resistance and strain characterisation

WGS-analysed isolates were tested for resistance to ampicillin, streptomycin, sulphonamides, tetracycline, gentamicin, chloramphenicol, enrofloxacin and cefotaxime by the disk diffusion susceptibility test [29]. The presence of antimicrobial resistance genes (ARG) was investigated in silico using the ResFinder database [30]. Mutations of *gyrA-gyrB* genes conferring resistance to quinolones were investigated by mapping the reads of the *gyrA* and *gyrB* genes of all sequenced isolates on *gyrA* and *gyrB* nt sequences of sensitive *S.* Typhimurium LT2 [31]. The presence of Insertion Sequence 26 (IS26), known to drive genomic evolution linked to the monophasic character of 4,[5],12:i:- strains [32], was investigated in the study genomes. To this end, sequencing reads were mapped on the biphasic *S.* Typhimurium LT2 reference genome (accession number AE006468) and on the IS26 composite transposon regions of the previously characterised monophasic strains VAR-2009/08643/1 (accession number K999732) [32], 07–2006 (accession number KR856283) [11] and 105/7/03 (accession number HQ331538) [33]. To further explore the genomic differences between pulsotype STYMXB.0131 and its single-band variant STYMXB.0083, the SNPs exclusive to the STYMXB.0083 pulsotype were extracted and annotated using Prokka 1.7 [34]. Exclusive SNPs were defined as those present in all the genomes of a pulsotype and absent in all other genomes of the study.

## Results

### Descriptive epidemiology

**Figure 1:**
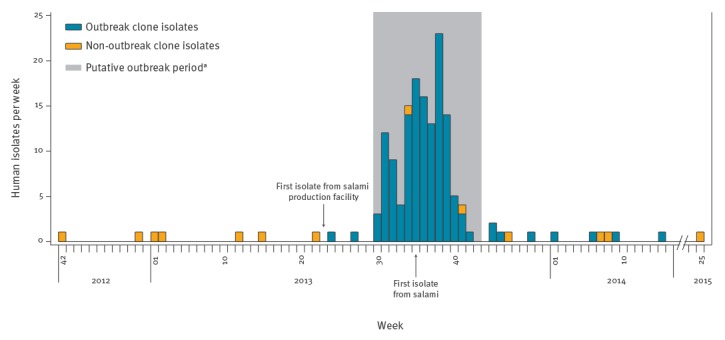
Number of isolates per week of *Salmonella* 4,[5],12:i:- pulsotype STYMX.0131 in Piacenza province, Italy, 2012–2015 (n = 158)

^a^ The putative outbreak period was defined based on the epidemiological evidence available.

The regional outbreak detection system signalled a potential outbreak by *Salmonella* 4,[5],12:i:- with pulsotype STYMXB.0131 on 14 August 2013. The first isolates with the outbreak pulsotype (OP) that were included in the alert by the signalling algorithm dated back to 22 July 2013. A total of 29 isolates with OP (each isolate corresponding to a different patient) had been recovered from the region between 22 July and 14 August. All but four of the isolates were from the province of Piacenza; therefore, it was considered that the potential outbreak concerned only that province, where the OP was increasing sharply while its incidence remained at the endemic level in the rest of the region where, overall, the OP was the most frequently reported pulsotype. The epidemic curve based on the case definition was steeply increasing, confirming the existence of the outbreak (Figure 1).

In particular, the average weekly number of isolates with the OP from Piacenza changed from 0.2 before the 22 July to 7.6 in the period 22 July to 14 August. The health authority was informed of the potential outbreak on 16 August. The interviews to the cases and their geographic restriction to a limited area of the province (in the north) rapidly led to the identification of a local facility producing fermented dry-cured salami made from pork as the possible source of the outbreak. The retail network of the producer overlapped with the location of the cases and the shop at the facility was mentioned repeatedly in case interviews. Microbiological confirmation of the facility as the source of the outbreak, interruption of distribution and recall of contaminated salami led to the reversion to the normal incidence of isolation of *Salmonella* 4,[5],12:i:- with OP and the outbreak was declared over on 29 October. Overall, 137 human isolates with OP were recovered from the outbreak territory between 22 July and 29 October. Based on the epidemiological evidence available, this time interval was identified as the putative outbreak period for the purpose of this study.

### Trace-back and environmental investigation

Inspection and microbiological investigation of the salami production facility demonstrated *Salmonella* 4,[5],12:i:- with OP in five of 21 tested batches of salami produced from 30 April to 10 September 2013 (representative isolates were: STM86; STM121; STM139; STM142; STM143; STM200). No batches processed before 30 April were still available for microbiology at the time of first inspection on 30 August. All environmental swabs (n = 50) were negative for *Salmonella*. The supply-chain information of the facility showed that some of the pork for salami production came from a small internal slaughterhouse at the facility and some was purchased from external industrial slaughterhouses. The pigs for the internal slaughterhouse originated predominantly from a single farm and to a lesser extent from other farms which supplied pigs occasionally.

Twenty-five faecal pools of 58 tested from the pen floors of the main pig-supplying farm were positive for *Salmonella* 4,[5],12:i:-, one had OP (STM204), all others had pulsotype STYMXB.0083, highly similar to the OP (representative isolates were: STM184; STM188; STM192; STM194; STM225; STM226). Pulsotype STYMXB.0083 was also detected on carcasses tested at the internal slaughterhouse and derived from pigs from that same farm (STM222; STM223; STM224). The facility’s HACCP records reported that a carcass swab from the internal slaughterhouse had been found positive for *Salmonella* before outbreak onset, on 3 June 2013. Upon typing, the isolate showed a *Salmonella* 4,[5],12:i:- with OP (STM40). The carcass was from one of the farms which supplied pigs occasionally. The microbiological sampling of this farm (six environmental boot swabs) remained negative. As a result of the investigation and follow-up monitoring, *Salmonella* with OP was further detected. It was isolated from four of 22 healthy workers at the facility (STM133; STM134; STM135; STM172), from a batch of meat that had not yet entered salami processing in the internal slaughterhouse on 23 September (STM156) and from two batches of salami from the facility in January and March 2015 (STM218; STM228). Remarkably, one of the industrial slaughterhouses supplying meat to the facility had reported the presence of *Salmonella* 4,[5],12:i:- with OP (STM22) during its internal routine testing of carcass surfaces, before outbreak onset, on 28 June 2013.

### Molecular epidemiology

**Figure 2:**
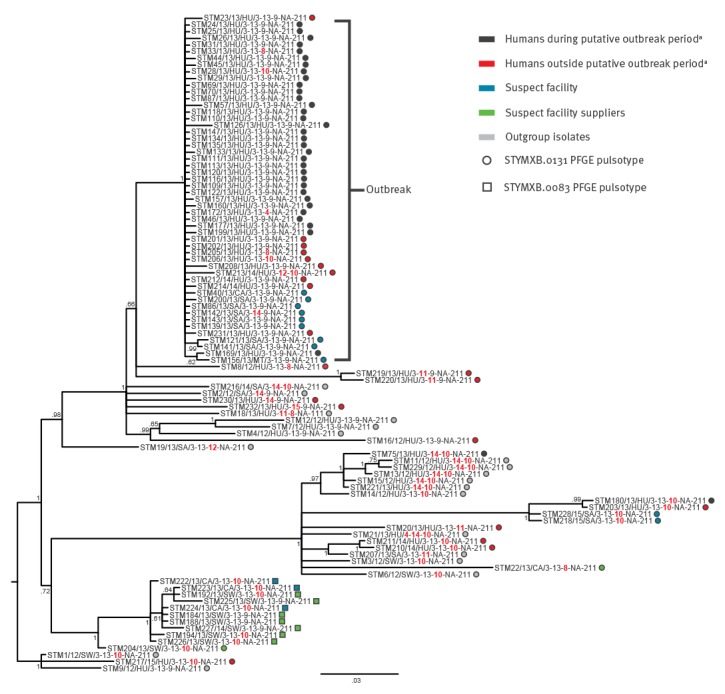
Phylogenetic Bayesian tree of *Salmonella* 4,[5],12:i:- isolates sequenced in this study, Emilia-Romagna, Italy, 2012–2015

CA: carcass; HU: human; MLVA: multilocus variable-number tandem repeat analysis ; MT: meat; SA: salami; SW: swine.

^a^ The putative outbreak period was defined based on the epidemiological evidence available.

All isolates included in the study had pulsotype STYMXB.0131 (part of the case definition) or its highly similar variant STYMXB.0083. As regards MLVA types, profile 3–13–9-NA-211 was detected in 132 isolates of 137 human isolates from the putative outbreak period, and was thus the most represented, but four other profiles were present among the remaining five isolates (STM28; STM33; STM75; STM172; STM180). Considering all human isolates eventually assigned to the outbreak cluster by WGS, four MLVA variants to 3–13–9-NA-211 were detected, representing six isolates (STM28; STM33; STM172; STM205; STM206; STM213) (Figure 2).

The string identifying each isolate reports the study ID of the isolate, the year of isolation, the origin and MLVA profile (3variant loci to the outbreak profile are in red).

The main outbreak profile (3–13–9-NA-211) was also detected among the isolates from salami, the internal slaughterhouse, the main pig-supplying farm and some of the outgroups of the phylogenetic analysis. Similarly to the human isolates, the isolates from the food chain showed a variety of MLVA profiles, generally closely related to profile 3–13–9-NA-211, consisting of single-locus variants most of the time (Figure 2).

### Whole genome sequencing

WGS analysis was performed to elucidate the individual responsibility of suspect sources along the food chain and to determine whether an outbreak-specific clone existed within the endemic STYMXB.0131 and what its time extension was. This high-resolution approach was required, given the existence of MLVA and PFGE variants among the outbreak isolates.

The outcome of the phylogenetic analysis is represented in Figure 2. Thirty-three of the 35 human isolates of the putative outbreak period, included in the analysis, belonged to the same clade that included the isolates from the four positive workers, all the isolates from salami collected during the investigation and the isolates from the internal slaughterhouse, STM40 (carcass before outbreak onset) and STM156 (meat for salami processing). No other isolates belonged to that clade except 10 humans out of the 21 recovered from Piacenza outside the putative outbreak period; the remaining 11 belonged to various different clades and were clearly not part of the outbreak. Seven SNPs were exclusive to and shared by all the outbreak isolates, supporting their cluster (Table 2).

**Table 2:** Single nucleotide polymorphisms exclusive to the outbreak clone (detected in all the outbreak isolates and in no other isolate of the study), Emilia-Romagna, Italy, 2012–2015

Position in reference	Non-outbreak Nt	Outbreak Nt	Codon change	Aminoacid change	Strand	Type of SNP	Gene	Product name
1005978	A	G	ATT → ATC	I → I	-	Synonymous	-	Short-chain dehydrogenase
1226252	A	G	TTG → CTG	L → L	-	Synonymous	mdtH	Multidrug resistance protein
2145307	C	T	ACT → ATT	T → I	+	Non-synonymous	pduX	L-threonine kinase
2265999	T	C	AAT → GAT	N → D	-	Non-synonymous	yehU	Sensor histidine kinase
3526676	A	G	TAC → TGC	Y → C	+	Non-synonymous	acuI	Putative acrylyl-CoA reductase
3912784	T	C	TGC → CGC	C → G	+	Non-synonymous	recG	ATP-dependent DNA helicase
4510357	A	C	TGG → GGG	W → G	-	non-synonymous	fdhF 1	Formate dehydrogenase

ATP: adenosine triphosphate; Nt: nucleotide; SNP: single nucleotide polymorphism.

Inside the outbreak clade, the number of SNPs to the consensus ranged from none to five (median value: one SNP), while the closest non-outbreak isolate (STM8) differed by 19 SNPs. As regards the responsibility of the food-chain operators, WGS confirmed the role of the salami producer, while both the main pig-supplying farm and the external slaughterhouse appeared to have no role in the contamination, different to what PFGE and MLVA testing had seemed to point to. In fact, all isolates with pulsotype STYMXB.0083 which came from the farm belonged to a well-segregated cluster in the phylogeny, distant from the outbreak clade (114 SNPs differed between the outbreak and the STYMXB.0083 consensus sequences), regardless of their PFGE and MLVA relatedness to the outbreak type. Similarly, the STYMXB.0131 isolates from the farm (STM204) and the external slaughterhouse (STM22) were shown to be distant from the outbreak clade. Remarkably, the isolates with OP from the salami facility of the follow-up monitoring were not related to the outbreak clone.

The phylogenetic analysis highlighted the clonality of the outgroup isolates from the 2012 nursery school outbreak, confirming the resolution power and accuracy of the analysis.

### Multilocus variable-number tandem repeat analysis variant analysis

**Figure 3:**
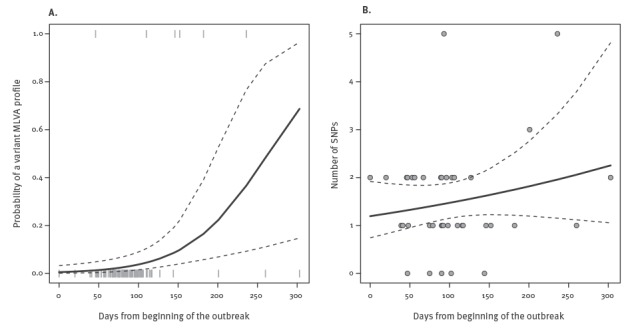
Probability of occurrence of variant multilocus variable-number tandem repeat analysis profiles (panel A) and single nucleotide polymorphisms to the consensus (panel B) in outbreak isolates as a function of the isolation date

MVLA: multilocus variable-number tandem repeat analysis; SNPs: single nucleotide polymorphisms.

Statistical analyses revealed that the probability of an outbreak isolate being an MLVA variant increased at the end of the outbreak (GLM output: chi-squared = 8.04, DF = 1, p = 0.0046), while the number of SNPs to the consensus did not significantly increase throughout the outbreak (GLM output: chi-squared = 1.65, DF = 1, p = 0.199), see Figure 3.

Generalised linear models with binomial distribution (panel A) or Poisson distribution (panel B). Solid black lines represent the best fit, dashed black lines represent 95% confidence intervals. In panel A, grey bars represent the occurrence (top bars) or not (bottom bars) of a variant MLVA profile in the isolate. In panel B, grey dots represent the number of SNPs to the consensus in the outbreak isolates.

### Antimicrobial resistance and strain characterisation

All sequenced isolates were tested for antimicrobial resistance and all but four were multidrug-resistant with R-type ASSuT (i.e. resistant to ampicillin, streptomycin, sulphonamides and tetracycline). The four exceptions did not belong to the outbreak clade and had ASSu R-type (STM2, STM3, STM232) or SuT R-type (STM8). The following ARGs were identified in all ASSuT isolates: *blaTEM* (ampicillin), *strA* and *strB* (streptomycin), *sul2* (sulphonamides), *tet*(*B*) (tetracyclines). The three ASSu isolates lacked *tet*(*B*) and the SuT isolate lacked *blaTEM* and *strB.* No mutations associated with quinolone resistance were detected in *gyrA* and *gyrB* genes of the sequenced isolates. Similarly to other 4,[5],12:i:- strains [11,32,33], both STYMXB.0131 and STYMXB.0083 isolates of the study possessed IS26 composite transposon insertions. Mapping of the sequencing reads on the LT2 reference genome revealed the deletion of STM2760-STM2772 chromosomal region in all sequenced genomes. This deletion, associated with IS26 insertions, corresponds to the loss of *fljAB* operon and *hin* gene (STM2770–2772 loci of LT2), the genetic determinants of biphasic phenotype. The loss was consistent, in all study isolates, with the negative *fljB-*targeted PCR [35]. Further analysis of IS26 transposon insertions was done through comparison with the IS26 composite transposon regions of the previously characterised monophasic strains VAR-2009/08643/1 [32], 07–2006 [11] and 105/7/03 [33]. The comparison confirmed STM2760-STM2772 deletion, as already observed in 07–2006, a strain with STYMXB.0131 pulsotype, where it was associated with the IS26-driven insertion of the RR3 element, a chromosomal module comprising various plasmid-derived genes and the ARGs *blaTEM*, *strA, strB*, *sul2* and *tet*(*B*). Conversely, the study genomes did not have the STM2753-STM2759 deletion observed in 105/7/03, and they did not present the insertion of RR1 and RR2 elements around the *fljAB* operon described in VAR-2009/08643/1. Furthermore, all genes reported in the RR3 element of strain 07–2006 were detected in the study genomes with only five exceptions. These were represented by non-outbreak isolates lacking some of the RR3 genes, namely from *tniAΔ,* to *methΔ* genes (STM2, STM3, STM232); from *tniAΔ* to *merR* genes (STM20); from *tnp2RΔ* to *tnpB* and *strB* genes (STM8). Overall, these findings support the hypothesis that an RR3-like resistance element was present in STYMXB.0131 and STYMXB.0083 of the study in place of the STM2760-STM2772 region, similarly to strain 07–2006. This analysis showed that STYMXB.0131 and STYMXB.0083 from this study did not differ from one another as regards IS26-related genomic architecture and antibiotic resistance genes. Conversely, five synonymous SNPs located on a single gene, *dus3,* a putative tRNA-dihydrouridine synthase, were found to be exclusive to STYMXB.0083.

## Discussion

### Antimicrobial resistance and strain characterisation

The ASSuT R-type and resistance genes of the outbreak (OB) clone indicate its belonging to the clonal lineage of *Salmonella* 4,[5],12:i:- with pulsotype STYMXB.0131 and chromosomal multidrug resistance to ampicillin, streptomycin, sulphonamides and tetracycline commonly circulating in Europe [12]. The two related pulsotypes of the study showed the same genomic structure as regards the insertions of IS26, involved in their monophasic phenotype. That structure was already reported in Italian strains of pulsotype STYMXB.0131 collected between 2008 and 2012 [11], confirming the long-lasting circulation of this lineage in Italy. Nevertheless, according to WGS phylogeny, in our scenario, the minimum difference between the two PFGE profiles did correspond to great phylogenetic distance between the OB isolates (having STYMXB.0131 pulsotype) and the STYMXB.0083 isolates from the suspected farm. This distance is apparent from the tree topology of the study isolates and is substantiated by 114 SNPs of difference between the OB and the STYMXB.0083 consensus sequences.

### Outcome of the investigation

WGS analysis accurately identified the existence of an outbreak-specific clone within the endemic pulsotype STYMXB.0131 of *Salmonella* 4,[5],12:i:- in the region affected. The 3-year long WGS monitoring extended the time boundaries of the outbreak beyond the originally identified (putative) outbreak period. Its duration was widened by 6 weeks before the epidemiologically-defined onset as a consequence of the identification of two early OB isolates and by 6 months after the apparent conclusion with the identification of eight late OB isolates (Figure 1). While the suspected salami was confirmed as the vehicle of infection, similarly to previous reports [36], the origin of the contamination upstream from the salami-processing facility was not demonstrated. The responsibility of the facility suppliers, initially suspected based on PFGE and MLVA, was not confirmed by WGS. In the absence of a demonstrated external origin of the contamination, this could be speculatively attributed to the positive healthy workers of the facility, although their positive status could be either the cause or the consequence of the salami contamination, with no possibility of solving the ambiguity. An alternative possible cause of the outbreak could have been persistent contamination inside the facility by the outbreak strain that could have entered the facility at some time before the outbreak, established itself and eventually contaminated the food products, reaching a level high enough to generate the observed high incidence of infections. The hypothesis of persistence is consistent with the sporadic isolation of the outbreak clone inside the facility before (STM40) and during the outbreak (STM156). Regardless of the origin of the contamination, the very limited number of SNPs differentiating the isolates of the OB cluster and the lack of an evolutionary structure inside the cluster are indicative of a single, time-restricted source of the OB. This would be consistent with a sudden expansion of the contaminating clone inside the production premises, regardless of its origin (e.g. the facility environment, the raw meat or carrier workers). The in-depth cleaning of the facility, following the demonstration of its implication, probably led to the eradication of the strain from the processing plant, as confirmed by the negative results of the 2-year long post-outbreak monitoring on hundreds of samples. These results coincided with the rapid decline and the disappearance of the clone from the human population within 6 months of the official outbreak closure.

### Performance of typing methods

Genomic investigation of the study outbreak through WGS confirmed, in a field scenario, that the high resolution of this approach allowed for the accurate assignment of the isolates to the outbreak clone, unlike current routine methods, PFGE and MLVA, which showed inadequate resolution and doubtful stability, respectively. This advantage of WGS, observed with a clonal pathogen like *Salmonella* 4,[5],12:i:- on a local geographical scale, could likely be helpful with clonal pathogens on larger geographical and temporal scales as well, where a greater number of variants can be expected with traditional typing methods. The use of WGS in widespread outbreaks, e.g. internationally, would imply sharing of standardised WGS data and analysis. In this study, MLVA showed a diversity of variants among the outbreak isolates that would potentially lead to incorrect assignment of some of the cases to the outbreak and to identify wrong links with suspected sources actually not related to the outbreak. This was the case for profiles 3–13–8-NA-211 and 3–13–10-NA-211 shared by some cases and suspected sources, which were eventually cleared of any responsibility. Even more misleading was the finding in the suspected pig farm of isolates having the main outbreak profile 3–13–9-NA-211 (STM184; STM188; STM225) which were eventually demonstrated not to belong to the outbreak clone. The results of other studies, conducted in recent years [8], and references thereof [37-39], are consistent with the ambiguity of MLVA evidenced by our data, despite the efforts of these authors to establish interpretation cut-offs for MLVA in terms of acceptable numbers of intra-outbreak repeats or variable loci. The high number of isolates analysed in our study and the complexity of the outbreak setting have shown limits of MLVA usage that those previous studies could not fully demonstrate. Interestingly, in our scenario the probability of emergence of MLVA variants was higher in the late phase of the outbreak, possibly reflecting mutation of the assayed loci with passing time at a rate higher than the rest of the genome, as measured through the accumulation of SNPs. While this is most likely not a limit in small and time-focused outbreaks, it could constitute a problem in lengthy ones.

## Lessons learnt

The primary origin of the outbreak clone or its introduction into the region remained unexplained. However, the observed appearance and disappearance in the human population of the outbreak clone during a 3-year monitoring, its association with a specific food plant and the absence of identified primary sources, tell us that the emergence of a clone of *Salmonella* capable of generating a significant outbreak can indeed be a time-limited, transient event, possibly caused by extemporary clonal expansions inside food processing facilities, not necessarily with substantial quantitative contributions by upstream primary production.

From the methodological point of view, the study case showed that, based on WGS data, the first isolates unambiguously linking the salami facility (STM40) and the infection of humans (STM231) were available on 3 June and 13 June, respectively. This was more than a month in advance of the outbreak onset based on incidence (22 July) and more than 2 months in advance of the identification of the salami facility as the source of contamination (beginning of September). Retrospectively speaking, the availability of such genomic evidence in real-time or within the few days needed to produce it, would have favoured the adoption of mitigation measures with enough advance to prevent dozens of infections, indicating the preventive value of WGS if used routinely and promptly in surveillance systems combing human and food isolates. Although the identification of epidemiological links between clinical and food isolates will continue to be central in the attribution of outbreaks to their sources, the availability to the food control authorities of ‘routine’ food-human matching information can contribute to better planning and prioritisation of the control activity on facilities and food chains. These results contribute to a better understanding of the dynamics of *Salmonella* contamination at the food-human interface and highlighted the potential of WGS to improve the procedures of surveillance, investigation and trace-back of *Salmonella* as observed by others [10,37,40-42].
